# Printing words in alternating colors facilitates eye movements among young and older Chinese adults

**DOI:** 10.3758/s13423-024-02581-6

**Published:** 2024-10-07

**Authors:** Jinger Pan, Aiping Wang, Mingsha Zhang, Yiu-Kei Tsang, Ming Yan

**Affiliations:** 1 Department of Psychology, The Education University of Hong Kong, Ting Kok, Hong Kong, China; 2 Faculty of Psychology, Beijing Normal University, Beijing, China; 3 State Key Laboratory of Cognitive Neuroscience and Learning and IDG, Division of Psychology, McGovern Institute for Brain Research, Beijing Normal University, Beijing, China; 4 Department of Education Studies, Hong Kong Baptist University, Hong Kong, China; 5 Centre for Learning Sciences, Hong Kong Baptist University, Hong Kong, China; 6 Department of Psychology, University of Macau, Macau, China; 7 Center for Cognitive and Brain Sciences, University of Macau, Macau, China

**Keywords:** Aging, Reading, Chinese, Word boundary

## Abstract

It is well known that the Chinese writing system lacks visual cues for word boundaries, such as interword spaces. However, characters must be grouped into words or phrases for understanding, and the lack of interword spaces can cause certain ambiguity. In the current study, young and older Chinese adults’ eye movements were recorded during their reading of naturally unspaced sentences, where consecutive words or nonwords were printed using alternating colors. The eye movements of both the Chinese young and older adults were clearly influenced by this explicit word boundary information. Across a number of eye-movement measures, in addition to a general age-related slowdown, the results showed that both groups benefited overall from the explicit color-based word boundary and experienced interference from the nonword boundary. Moreover, the manipulations showed stronger effects among the older adults. We discuss implications for practical application.

Reading is one of the most important life skills. During the reading of continuously written text, readers’ eyes move along the words to obtain visual and linguistic information during fixations when the eyes remain relatively still. Over the past decades, research on eye movements during reading has almost extensively focused on skilled adults reading alphabetic scripts, highlighting that fixation location and duration are among the most reliable indices for lexical processing in natural sentence reading (Kliegl et al., 2006; Rayner, 1998). Recently, a growing body of research has revealed cognitive processes and oculomotor activities in other writing systems and how they develop across the lifespan. One distinct visual feature of the Chinese script is the lack of interword spaces, offering an opportunity to examine and extend psychological theories of lifespan development from a cross-script comparison perspective. Here, we present a study showing that printing words in alternating text colors can facilitate young and older Chinese adults’ natural sentence reading. However, in this study, the older adults benefited more from such color-based word boundaries and, at the same time, experienced more interference from inconsistencies between perceptually and semantically grouped characters.

The interword spaces are important cues for readers of spaced scripts to guide their eyes through continuous text. Using distinct low-spatial-frequency information, readers can segment an upcoming word visually and target its center as their intended fixation location for optimal lexical processing (O’Regan et al., 1984). Due to systematic and random oculomotor errors, first-fixation location (FL; where eye gaze initially lands within a word) distribution typically forms a Gaussian distribution peaking around the word center, suggesting that word length plays a primary role in eye guidance (Rayner, 1979). In contrast, several Asian scripts, such as Chinese, Japanese, and Thai, are written naturally without visual clues for word boundaries, therefore raising radical challenges to all eye-movement theories and models. First, is word-based saccadic programming a universal mechanism in reading? Second, if so, how do readers of unspaced scripts achieve word segmentation independent of interword spaces? Finally, do readers benefit from artificially introduced word boundaries? Below, we review the relevant literature from these perspectives.

Recent years have witnessed a rapid development of eye-movement research in Asian languages, especially in Chinese (Reilly & Radach, 2012; Siegelman et al., 2022). Indeed, these abovementioned challenges have been the center of controversy in Chinese reading research. Crucially, characters need to be grouped into larger units for correct understanding. At a character level, one single character by itself often indicates multiple morphemes. Multicharacter compound words can provide a minimal context to determine the correct morpho-semantic meanings (e.g., Tsang & Chen, 2010). For instance, the character 花 can mean *flower* or *to spend*. A concrete meaning can only be selected in words such as 花瓣 (*flower petal*) or 花钱 (*to spend money*). At a phrase level, over 80% of Chinese characters can involve word boundary ambiguity (Yen et al., 2009). Figure 1 illustrates an example of a character string parsed with different word boundaries, leading to different meanings.

**Fig. 1:**
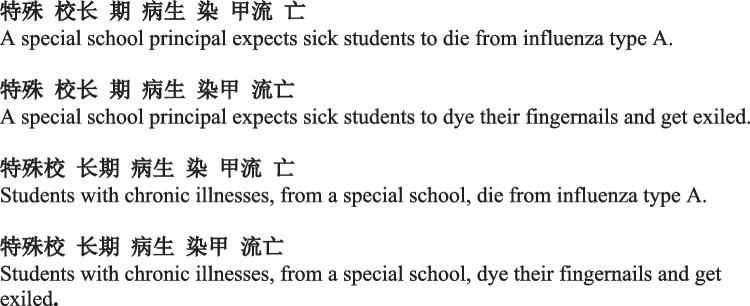
Four different ways to parse and understand a string “特殊校长期病生染甲流亡”, the title of a newspaper report from *Ming Pao* newspaper, with spaces to indicate word boundaries and translations under each sentence

Different saccadic programming accounts have been proposed concerning when and where word segmentation occurs. Yan et al. (2010) hypothesized a dynamic model in which saccadic programming follows a parafoveal word segmentation process. Chinese readers target the center of the upcoming word when it has been segmented during prior fixations. Otherwise, a character-based saccade towards the first character beyond the current word is generated. In a similar vein, Fan and Reilly (2022) proposed character-based eye guidance for long incoming saccades and word-based eye guidance for short ones, presumably because word segmentation is more likely to fail when a saccade is launched farther away (Yan & Kliegl, 2016). In contrast, the processing-based model assumes that a saccade targets the next unprocessed character during each fixation (Liu et al., 2015), advocating a relatively late, foveal word segmentation. The two accounts, nevertheless, agree that word segmentation is a critical step during reading.

Considering the importance of explicit word boundaries, researchers have hoped to benefit reading by introducing interword spaces to unspaced scripts. However, empirical results have shown rather mixed evidence. In some studies, typical Chinese readers benefited from interword spaces only for ambiguous and difficult sentences but not for normal ones (Bai et al., 2008; Hsu & Huang, 2000a, 2000b; Liu et al., 1974). Kasisopaa et al. (2013) found that reading was largely unaffected when inserting interword spaces in the Thai language. Arguably, artificially inserted interword spaces may interfere with readers’ well-established unspaced reading (Kohsom & Gobet, 1997). Similarly, in Japanese studies, reading benefits appeared only for sentences written purely with kana characters, possibly because the visually distinct kanji characters can already indicate word boundaries (Kajii et al., 2001; Sainio et al., 2007). Meanwhile, skilled readers of these unspaced scripts rely on other information to compensate for the lack of interword spaces. For instance, Yen et al. (2012) proposed that skilled Chinese readers use statistical knowledge to segment words. Compared with visual cues in spaced scripts, an important prerequisite for such statistics-based word segmentation is that the reader has adequate vocabulary knowledge. Correspondingly, the development of Chinese children’s eye guidance lags behind that of their English counterparts. English-speaking first-graders already exhibit adult-like saccadic patterns (McConkie et al., 1991). In contrast, Chinese children show a late maturity in eye guidance: Dyslexic fifth-graders have been found to generate more character-based saccades than their typically developing peers (Pan et al., 2014), and even typically developing fifth-graders’ FL has been seen as less accurate than that of adults (Yan et al., 2019).

Recently, color-based perceptual grouping has been introduced to provide a visual cue for syllabic and word boundaries while preserving unspaced text layout. Häikiö et al. (2015) tested Finnish first- and second-graders and reported shorter fixations during reading when polysyllabic words were presented with alternating text colors for syllables than when they were hyphenated at syllabic boundaries. Similarly, Perea et al. (2015) showed that using alternating text-color words could compensate partly for the removal of interword spaces during Spanish reading. In the Chinese language, Perea and Wang (2017) extended this “less intrusive visual cue than inserting interword spaces” (p. 1165) and reported faster oral reading speed in colored text than normal text among second-graders and adults. Zhou et al. (2018) designed a new colored nonword condition, whereby characters from different words were printed in the same color. They found oculomotor benefits in the word segment condition and costs in the nonword segment condition over the mono-color baseline. Follow-up studies with third-graders (Pan et al., 2021) and second-language learners of Chinese (Zhou et al., 2020) showed robust facilitation due to word segmenting.

From a lifespan perspective, it is of great practical importance to improve reading performance in another vulnerable group of the population, the older adults. Older adults suffer from reduction in visual acuity to perceive details, implying a smaller perceptual span in reading (i.e., the area of effective vision during a single fixation; McConkie & Rayner, 1975). Indeed, older adults obtain less information about upcoming words than do young adults (Rayner et al., 2009, 2010, 2014). Risse and Kliegl (2011) reported that young German adults’ fixations were influenced more by the properties of upcoming words than were older adults. Although aging typically leads to prolongation of fixation in reading alphabetic scripts, saccadic programming, as reflected by FL was, however, unaffected by age (Paterson et al., 2013; Rayner et al., 2006). For instance, Rayner et al. (2006) reported that their young readers tended to fixate slightly further than the older readers on the target word, but both groups’ FLs were close to the word center. Additionally, the FL difference was likely caused by the older readers’ further launch sites. Moreover, refixation probability was not statistically different for the two groups. Like English-speaking children, older adults possibly make good use of spatial frequency contrasts afforded by interword spaces to select saccade targets easily. Supporting this view, McGowan et al. (2014) found that older readers’ eye movements were more severely disrupted by the removal of interword spaces than was the case with young adults, particularly when the spaces were replaced by open squares as they included features similar to linguistic symbols. The authors concluded that older readers make use of coarse visual information to guide their eye movements. In contrast, aging effects in FL have been shown in Chinese. For instance, older Chinese adults fixated more often than young adults near the word beginnings (Wang et al., 2018) and made fewer single fixations (i.e., a word is inspected with exactly one fixation during its first-pass reading; Li et al., 2018). Considering that single-fixation cases are a likely consequence of successful target-word segmentation, whereas FLs further away from the word centers presumably indicate target-word segmentation failure (Yan et al., 2010), the results presented above suggest that older Chinese adults’ reading difficulties also lie in saccadic programming. Possibly, older Chinese adults may encounter specific difficulties with word segmentation because of their inefficient parafoveal processing caused by visual decline.

The present study was undertaken to follow up on prior research and investigate whether word boundary information afforded by text color would assist older Chinese adults during their reading of naturally unspaced sentences. Our predictions were as follows. Considering that typical visual degeneration can prevent older Chinese adults from perceiving adequate information for word segmentation, explicit word boundaries may facilitate eye movements more for older adults than for young ones. As well, older adults should be affected more by interference from inconsistent information between perceptually and semantically grouped characters in the nonword segment condition.

## Method

### Participants

Sixty older adults (*M*_age_ = 66.0 years, *SD* = 3.6, 41 women) and 59 young adults (*M*_age_ = 23.0 years, *SD* = 2.1, 35 women), who were native Chinese speakers with normal or corrected-to-normal vision, participated in the eye-tracking experiment. All participants had completed high school (i.e., at least 11 years of formal education). None of the participants reported any history of color-blindness. The sample sizes of the participant groups and the reading materials were determined following recent simulation work by Kumle et al. (2021), who recommended samples of 60 participants and 120 sentences for adequate statistical power in corpus analyses using linear mixed models (LMMs). In accordance with the Declaration of Helsinki, the experimental procedures were approved by the Ethics Committee of Beijing Normal University. The participants gave their written consent before commencing the test. None of the participants had been diagnosed with any attentional, color-related, or reading-related deficits.

We measured all participants’ raw eye visual acuity. Based on the right eye (the recorded eye during the eye-tracking experiment), the young adults’ visual acuity (*M* = 1.10, *SD* = 0.28, corresponding to roughly 20/18 in the Snellen ratio) was higher than that of the older adults (*M* = 0.85, *SD* = 0.26, corresponding to 20/25 in the Snellen ratio), *t*(117) = 4.970, *p* < 0.001. The participants wore glasses, if needed, to reach corrected-to-normal vision during the eye-tracking experiment. We tested the vocabulary knowledge and short-term memory of the two groups using the vocabulary and digit span tasks in WAIS-RC (Gong, 1982) and extracted the scaled scores for individual participants based on their ages in these two tests. The older adults (*M* = 16.27, *SD* = 0.63) had better vocabulary knowledge than the young adults (*M* = 15.44, *SD* = 0.62), *t*(117) = 7.16, *p* < 0.001. Similar to previous aging studies (e.g., Risse & Kliegl, 2011; Zhao et al., 2019), the young adults (*M* = 15.58, *SD* = 2.33), however, performed better than the older adults in the digit span task (*M* = 13.10, *SD* = 2.40), *t*(117) = 5.715, *p* < 0.001.

## Material and design

**Fig. 2:**
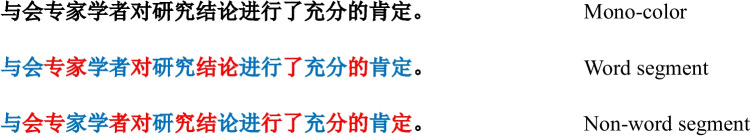
An illustration of experimental stimuli in different conditions. The sentence is translated as: “Experts and scholars attending the meeting fully affirmed the research conclusions”

We adopted a 2 (age group: young and older adults) × 3 (text color: mono-color, word segment by color, and nonword segment by color) design. In the baseline mono-color condition, all words in the sentences were presented normally, colored black. In the other two conditions, alternating text colors were used to match word boundaries in the word segment condition, or to group characters from different words in the nonword segment condition (see Fig. 2 for illustration). We designed the materials carefully so that the numbers of segments in the word segment condition (*M* = 11.23, *SD* = 1.57) and in the nonword segment condition (*M* = 11.36, *SD* = 1.74; *p* > 0.1) did not differ. The stimuli for the eye-tracking experiment were the 150 sentences from the Beijing Sentence Corpus (Yan et al., 2010). The sentences in this corpus were selected from the *People’s Daily*, an official newspaper of the People’s Republic of China, and edited slightly to remove possible semantic and word-boundary ambiguities. Further details of the corpus, such as word frequency, visual complexity, length, and predictability information, were reported by Pan et al. (2022). The experimental sentences were presented in a randomized order for each participant, with a constraint that no more than three consecutive sentences were of the same condition.

## Apparatus

Eye movements were recorded with an EyeLink 1000 system running at 1000 Hz. Each sentence occupied only one line on the screen of a 22-in. NEC-Mitsubishi FE2111SB monitor (resolution 1,280 × 1,024 pixels; frame rate 105 Hz). The participants were seated comfortably with a forehead-and-chin rest 70 cm from the monitor. We used Song Font, and each character horizontally occupied 48 pixels and subtended 1.2° of visual angle. All recordings and calibrations were done monocularly based on the right eye, and viewing was binocular.

## Procedure

Before the eye-movement recording started, the participants’ gaze positions were calibrated with a 5-point grid (maximum error < 0.5^°^). Prior to the presentation of each sentence, a drift checkpoint appeared on the left side of the monitor. The participant’s gaze on the point initiated the presentation of the next sentence, with its first character occupying the position of the point. Otherwise, if the eye tracker failed to detect the gaze around the point, a recalibration was performed. The participants were instructed to read the sentences silently for comprehension, then to fixate on a dot in the lower-right corner of the monitor, and finally to press a keyboard button to signal the completion of a trial. They were given 15 practice trials before their reading of the experimental sentences. Thirty-eight sentences were randomly selected, each to be followed by an easy yes–no comprehension question to encourage the participants’ engagement. The young and older participants correctly answered 94.4% (*SD* = 4.4%) and 84.0% (*SD* = 6.4%) of the questions, respectively.

## Data analysis

Fixations were determined with an algorithm for saccade detection introduced by Engbert and Kliegl (2003). Sentences were removed due to participants’ blinks, coughs, or body movements during reading, or to tracker errors (*n* = 2,251; 12.6%), and also due to extremely low numbers of fixations (i.e., fewer than one third of words were fixated on, *n* = 17; 1.0%). In addition, for the remaining sentences, the first and last words and the first and last fixated words in each trial were removed. Words with first-fixation durations (FFDs; duration of the first fixation on a word during first-pass reading, irrespective of the number of fixations) shorter than 60 ms or longer than 600 ms and gaze durations (GDs; the sum of fixation durations during the first-pass reading of a word) longer than 800 ms were excluded from the data analyses (*n* = 6,378; 4.7%). Because most of the fixated words (*n* = 77,315; 77.8%) were two-character words, we focused on these words for the data analyses to avoid confounding due to word length. The dependent variables included FFD, GD, global reading speed (RS; the number of characters processed per minute), first-fixation location (FL; landing position of the first fixation on a word relative to word beginning), total reading time (TRT; sum of all fixations on a word including regressive fixations), refixation probability (RfP; the likelihood of making an additional fixation on the word before moving forward) and regression probability (RgP; the likelihood of making a backward saccade). We used LMMs for the analyses of the continuous dependent variables and generalized linear mixed models (GLMMs) for the analyses of categorical data. Experimental effects were estimated using the lmer program of the lme4 package (Version 1.1–23; Bates et al., 2015) in the R environment for statistical computing and graphics (Version 3.6.3; R Core Team, 2018). The lmerTest package (Version 3.1–2; Kuznetsova et al., 2017) was used for the *p* values. The dependent variables of the viewing duration measures were log-transformed in the LMMs (Kliegl et al., 2010). For the fixed effects, we specified a *treatment contrast* with the mono-color condition as the reference, a *sum contrast* for the factor of age group, an interaction between these two factors, and a centered covariate of launch site. For the random effects, we started with models with subject- and item-related variance components for intercepts and random slopes for fixed effects, and reported parsimonious LMMs for successful convergence (Matuschek et al., 2017).

## Results

**Fig. 3:**
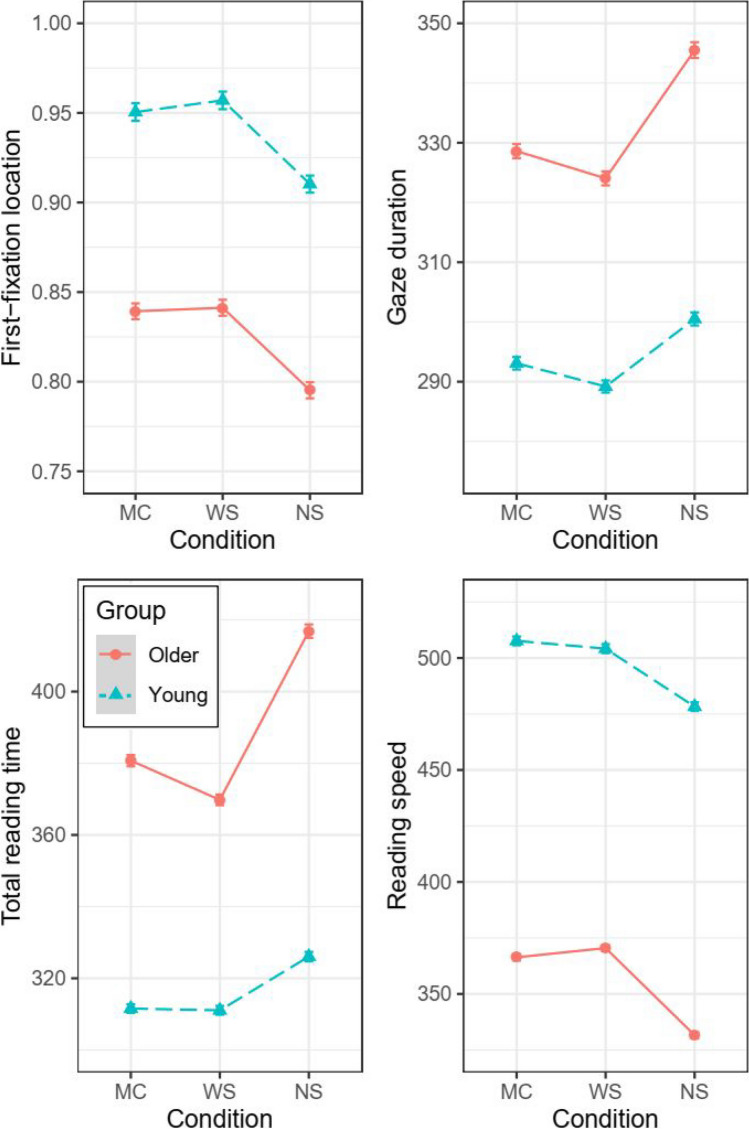
Experimental effects on first-fixation location (top-left panel), gaze duration (top-right panel), total reading time (bottom-left panel), and reading speed (bottom-right panel). Partial effects were plotted using the remef package (Version 0.6.10; Hohenstein & Kliegl, 2015). Graphics were generated using the ggplot2 package (Version 2.1.0; Wickham, 2009). MC = mono-color, WS = word segment, and NS = nonword segment

In a number of the oculomotor indices, both groups of participants demonstrated better performance in the word segment condition as compared with the mono-color baseline (conditional means in Table 1 and statistics in Table 2). They exhibited reductions in GD, TRT, and refixation probability. Furthermore, the nonword segment condition interfered with reading, as the participants slowed down their global RS, sent their fixations closer to the word beginnings, increased their viewing durations, and made more refixations and regressions. We also observed main effects of age group, showing that the older adults read more slowly, had their FLs closer to the word beginnings, fixated on words less briefly, refixated on words more often, and made more regressions (see Fig. 3).

**Table 1:** Reading measures

	RS	FL	FFD	RfP	GD	RgP	TRT
Older adults							
MC	375 (79)	0.83 (0.10)	265 (32)	26.5 (13.7)	332 (44)	10.7 (5.7)	387 (69)
WS	380 (79)	0.84 (0.10)	266 (31)	24.5 (13.2)	327 (45)	9.8 (4.8)	375 (66)
NS	342 (82)	0.80 (0.08)	272 (34)	29.2 (14.8)	349 (50)	12.5 (6.3)	427 (89)
Young adults							
MC	516 (103)	0.93 (0.08)	261 (35)	14.7 (10.5)	297 (43)	6.6 (4.4)	317 (50)
WS	513 (102)	0.94 (0.08)	259 (36)	14.1 (9.3)	293 (43)	7.1 (4.6)	316 (50)
NS	490 (108)	0.90 (0.09)	263 (38)	16.3 (11.0)	305 (45)	6.9 (4.4)	332 (57)

Means (and standard deviations in parentheses) of reading speed (RS) in characters per min, first-fixation location (FL) in character, first-fixation duration (FFD) in ms, refixation probability (RfP) in percentage, gaze duration (GD) in ms, regression probability (RgP) in percentage, and total reading time (TRT) in ms, for the three experimental conditions, split by age group. Values were computed across participants’ means. MC = mono-color; WS = word segment; NS = nonword segment

**Table 2:** Model outputs

	RS			FL			FFD			RfP			GD			RgP			TRT		
	Est	*SE*	*t*	Est	*SE*	*t*	Est	*SE*	*t*	Est	*SE*	*z*	Est	*SE*	*t*	Est	*SE*	*z*	Est	*SE*	*t*
(Intercept)	6.047	0.021	**288.67**	0.899	0.017	**52.36**	5.519	0.012	**455.79**	− 1.630	0.089	** − 18.23**	5.660	0.014	**403.71**	− 2.642	0.065	** − 40.65**	5.751	0.017	**334.85**
ls.c	NA	NA	NA	− 0.365	0.002	** − 172.30**	0.011	0.001	**7.32**	0.495	0.013	**38.53**	0.058	0.002	**30.77**	0.525	0.016	**33.64**	0.094	0.002	**46.62**
cwd	0.002	0.004	0.57	0.006	0.005	1.13	− 0.003	0.003	− 1.07	− 0.094	0.024	** − 3.96**	− 0.012	0.003	** − 3.53**	0.004	0.033	0.11	− 0.013	0.004	** − 3.49**
cnd	− 0.083	0.004	** − 21.15**	− 0.054	0.004	** − 13.82**	0.014	0.003	**5.00**	0.162	0.023	**6.99**	0.033	0.004	**7.84**	0.149	0.033	**4.56**	0.061	0.005	**11.49**
group	0.328	0.040	**8.20**	0.226	0.034	**6.75**	− 0.017	− 0.024	− 0.72	− 1.025	0.176	** − 5.81**	− 0.121	0.027	** − 4.43**	− 0.836	0.124	** − 6.75**	− 0.212	0.033	** − 6.46**
cwd × group	− 0.023	0.008	** − 2.87**	0.003	0.010	0.29	− 0.009	0.005	− 1.68	0.022	0.047	0.46	0.000	0.007	0.02	0.193	0.066	**2.92**	0.019	0.007	**2.55**
cnd × group	0.040	0.008	**5.17**	− 0.006	0.008	− 0.75	− 0.012	0.005	** − 2.21**	− 0.046	0.046	− 1.00	− 0.020	0.008	** − 2.36**	− 0.088	0.065	− 1.35	− 0.037	0.011	** − 3.52**

RS = reading speed, FL = first-fixation location, FFD = first-fixation duration, RfP = refixation probability, GD = gaze duration, RgP = regression probability, TRT = total reading time, Est. = estimate (regression coefficient), *S.E.* = standard error, ls.c = launch site (centered), cwd = contrast of word segment vs. mono-color, cnd = contrast of nonword segment vs. mono-color

Moreover, the interactions between the factors were reliable in RS and all fixation-duration measures. Simple effects revealed larger interferences due to inconsistent visual and semantic groupings of characters (i.e., the nonword segment vs. mono-color contrast) for the older adults (GD: *b* = 0.044, *SE* = 0.005, *t* = 8.434, *p* < 0.001; TRT: *b* = 0.080, *SE* = 0.005, *t* = 14.642, *p* < 0.001) than for the young adults (GD: *b* = 0.022, *SE* = 0.005, *t* = 3.840, *p* < 0.001; TRT: *b* = 0.040, *SE* = 0.005, *t* = 8.151, *p* < 0.001). As well, our results demonstrated benefits from the word segment condition over the mono-color baseline only for the older adults, showing increased RS (*b* = 0.013, *SE* = 0.006, *t* = 2.329, *p* = 0.20), reduced regression rate (*b* =  − 0.096, *SE* = 0.042, *t* =  − 2.277, *p* = 0.023), and TRT (*b* =  − 0.022, *SE* = 0.005, *t* =  − 4.047, *p* < 0.001). For the young adults, the differences between word segment and mono-color conditions were not significant (all *p* values > 0.05).

## Discussion

We adopted a manipulation of text color to indicate correct/incorrect word boundaries while maintaining the naturally unspaced visual layout of Chinese sentences. The explicit boundaries have been shown previously to facilitate readers’ eye movements and reading in different populations. We conducted the present study from a lifespan-development perspective. Due to a common reduction in visual acuity, reading can be challenging for older adults. In particular, Chinese is more densely written than many other scripts, inducing a greater disadvantage due to aging. Our results indicate that both groups benefited from the explicit word boundary, with larger gains for the older adults. Meanwhile, their oculomotor activities were delayed more severely due to inconsistent visual and semantic groupings of characters. Below, we discuss how word boundaries afforded by text color can improve reading and how eye guidance in Chinese reading possibly develops across one’s lifespan. This discussion is followed by some practical implications.

Inserting interword spaces into Chinese sentences, in the hope of increasing reading performance, goes back a long way but leads to rather mixed outcomes. In contrast, color-based word segmentation provides a less interfering visual cue. The benefit emerges from chromatic similarity within words, which induces highly automatic and unconscious perceptual grouping (e.g., Pinna et al., 2010), and has been shown consistently at behavioral, oculomotor, and neurological levels. For instance, exploring the neurological foundation of the effect in an fMRI study, Zhou et al. (2019) demonstrated that word segmentation by text color modulated the connections between the visual word form area (VWFA) and dorsal attention regions, and between the VWFA and language-related regions, confirming that the benefit arises from better perceptual grouping of characters.

Extending the effect of color-based word boundary cues to older Chinese adults, the current study has provided insights into aging effects on reading. Oculomotor control during reading involves two dynamic eye-movement decisions: when to move the eyes and where to send them, as reflected respectively by fixation duration and location. These decisions are largely independent of each other (e.g., Brysbaert et al., 2005; Vainio et al., 2009). Additionally, it has been established that different fixation measures are associated with different temporal stages during processing (Inhoff, 1984; Inhoff & Radach, 1998). As such, by analyzing a series of reading and oculomotor indices, we were able to identify the mechanisms underlying the observed effects of alternating color. In particular, both TRT and RgP are related to regressive eye movements, and therefore they are considered typically as second-pass reading measures to indicate a late processing stage. Notably, moving back to previously processed words for rereading, regression, is a costly behavior in terms of reading efficiency. Our results, therefore, suggest that the benefit of color-based word boundary cues lies in improving older adults’ global reading speed by reducing their rereading.

Some previous studies have shown facilitation in early saccade generation among other populations, as reflected by FLs shifting further away from the word beginnings (e.g., Zhou et al., 2018). Although the FL effect appeared in the same numerical direction among the older adults in the current study, they were of a much smaller magnitude and were not statistically significant. One possible explanation for the lack of FL benefits for the older adults could be their risky reading strategies (e.g., Rayner et al., 2006, 2009). Compared with young adults, they tend to generate longer saccades to compensate for reading speed but make regressions more often in case of wrong predictions of upcoming words. Even though there is still a need to consolidate whether Chinese older adults adopt the same reading strategy (Zhang et al., 2022), the provision of word boundary cues in the parafovea may nevertheless promote their prediction of word length and reduce their regression for resegmentation. Together, the significant interactions in TRT and RgP may reflect an age-related benefit during a higher-level information integration process, rather than initial saccade generation or lexical access that the segmentation cue is expected to facilitate.

Meanwhile, it is also worth noticing a number of processing costs that occurred in the nonword segment condition in both groups. Overall, incorrect word segmentation led the FLs closer to the beginning of words. In particular, the older adults were affected more severely by such an inconsistency between perceptually and semantically grouped characters. Therefore, saccade generation in Chinese can be influenced by exogenous visual cues from the word boundary. These results are compatible with the dynamic saccade model (Yan et al., 2010): Inconsistent groupings of characters at a low (i.e., perceptual) level and at a high (i.e., semantic) level possibly confuse readers’ saccade generation and reduce the likelihood of successful parafoveal word segmentation. In a broader view, our results agree with earlier findings of age-related decrements in Stroop test performance, that older adults show greater delays in responding to the ink color of a target word, when the meaning of the word is different from the ink color, leading to greater Stroop color-word effects (e.g., Cohn et al., 1984; Davidson et al., 2003). These results are considered to support age-related deficits in a cognitive inhibitory process (Hasher & Zacks, 1988) or can be attributed to a general slowdown (Verhaeghen & De Meersman, 1998). Wolf et al. (2014) combined diffusion tensor imaging (DTI) and behavioral results and concluded that cognitive inhibition, as reflected by the Stroop effect, declines with age.

As one of the most critical foundations for reading research, studies have shown that the size of the perceptual span is not constant but is influenced by various factors, including individual differences in reading proficiency and the visual properties of the script involved. The basic writing units are densely packed in Chinese, leading to a perceptual span of only one previous character and three or four upcoming ones (Inhoff & Liu, 1998; Yan et al., 2015). The age-related reduction in the perceptual span due to visual degeneration (Rayner et al., 2009, 2010, 2014; Risse & Kliegl, 2011) may pose an even greater reading difficulty for older readers of unspaced scripts than for those reading spaced scripts. Given the lack of visual cues for word boundary, the achievement of word segmentation is assumed to be based on statistical, rather than visual, information. Therefore, it is critical to word-boundary estimation to obtain adequate information from characters within the perceptual span. In addition, Li et al. (2018) reported a larger word length effect on fixation duration in Chinese, as their older participants made longer fixations than did the young adults on long words, concluding that older adults experience a higher level of difficulty in word segmentation. Against this background, it is reasonable to expect our older adults to derive greater benefits from explicit word boundaries. This hypothesis was supported by the significant interaction between the word segment versus mono-color baseline contrast and age group. The observed benefit of a reading speed of five characters/min for the older adults was not gigantic, possibly because our current sample showed relatively well-preserved cognitive abilities as reflected by their vocabulary knowledge and short-term memory test scores. It would be interesting to extend the paradigm to other older adult populations, such as those with minor cognitive disorders. In addition, given that reading is a frequent activity in daily life, a small difference in reading rate may lead to desirable benefits. After all, it is of theoretical interest to extend our knowledge about lifespan development in reading.

It is of theoretical and practical value to extend the study to other older Chinese populations. There are two written varieties of the Chinese script—namely, traditional (TC) and simplified (SC). It has been documented that visual complexity is one of the most reliable predictors of reaction time in isolated word recognition and fixation duration in sentence reading (see Tsang & Chen, 2012, for a review). SC characters are often written with fewer strokes, making them easier to read, especially at low screen resolutions and at low visual acuity. TC characters are used commonly as the official script in Taiwan, Hong Kong, and Macau. Due to the higher visual complexity of TC, older adults may encounter greater difficulty in perceiving characters than their SC counterparts who were examined in the current study. It is hypothesized, therefore, that older TC adults demonstrate a high reliance on visual cues for word segmentation.

To summarize, our results showed that word boundary afforded by text color facilitates oculomotor activities, especially among older adults. The study suggests that printing words in alternating colors could improve age-related declines in Chinese reading.

## Data Availability

The data and scripts for analyses are publicly available from the Open Science Framework from this link: https://osf.io/xndm4/

## Declarations

### Author note

This study is not preregistered.
